# Supplementary material for: Intertwined risk factors of mental health and cardiovascular diseases: A cross-sectional survey in Godawari Municipality of Far-western Nepal
Source: PLoS One. 2025 May 30;20(5):e0321436. doi: 10.1371/journal.pone.0321436 (PMC12124577; doi:10.1371/journal.pone.0321436)
Supplement: S1 File — (DOCX) [file pone.0321436.s002.docx]

S2.Table 1: Factors associated with depressive symptoms

| **Variable** | **Depressive symptoms** | | **χ²** | **p-value** |  |  |
| --- | --- | --- | --- | --- | --- | --- |
|  | **Presence [184 (47.2%)]** | **Absence [206 (52.8%)]** |  |  |  |  |
| **Socio-demographic** |  |  |  |  |  |  |
| **Age** |  |  | 18247.500^b^ | 0.511 |  |  |
| 30-39 years | 50 (43.1) | 66 (56.9) |  |  |  |  |
| 40-49 years | 45 (51.7) | 42 (48.3) |  |  |  |  |
| 50-59 years | 33 (45.8) | 39 (54.2) |  |  |  |  |
| 60+ years | 56 (48.7) | 59 (51.3) |  |  |  |  |
| Md(Q1~Q3) | 48(37~62) | 48(35~60) |  |  |  |  |
| **Sex** |  |  | 7.689 | **0.006**** |  |  |
| Male | 48 (37.2) | 81(62.8) |  |  |  |  |
| Female | 136 (52.1) | 125(47.9) |  |  |  |  |
| **Ethnicity** |  |  | 1.732 | 0.188 |  |  |
| Brahmin/Chhetri | 149(45.7) | 177(54.3) |  |  |  |  |
| Dalit, Janajati and Madhesi | 35 (54.7) | 29 (45.3) |  |  |  |  |
| **Marital status** |  |  | 0.233 | 0.630 |  |  |
| Never married | 1 (33.3) | 2 (66.7) |  |  |  |  |
| Ever Married | 183 (47.3) | 204 (52.7) |  |  |  |  |
| **Education** |  |  | 0.125 | 0.874 |  |  |
| Illiterate | 36 (48.0) | 39 (52.0) |  |  |  |  |
| Literate^@^ | 148 (47.0) | 167 (53.0) |  |  |  |  |

| **Occupation** | |  | |  | | 3.058 | | 0.217 |
| --- | --- | --- | --- | --- | --- | --- | --- | --- |
| Unemployed | | 45 (49.5) | | 46 (50.5) | |  | |  |
| Housekeeper, Farmer and Labor | | 122 (48.6) | | 129 (51.4) | |  | |  |
| Private employee, Government job and Business | | 17 (35.4) | | 31 (64.6) | |  | |  |
| **Monthly family income** | | | |  | | 18663.000^b^ | | 0.736 |
| ≤ 30000 NPR | | 133 (47.0) | | 150 (53.0) | |  | |  |
| >30000 NPR | | 51 (47.7) | | 56 (52.3) | |  | |  |
| Md(Q1~Q3) | | 30000  (25000~38750) | | 30000  (25000~40000) | |  | |  |
| **CVDs Risk Factors**  **Smoking status** | | | |  | | 0.721 | | 0.396 |
| Never or former smoker | | 156 (48.1) | | 168 (51.9) | |  | |  |
| Current smoker | | 28 (42.4) | | 38 (57.6) | |  | |  |
| **Number of cigarettes/days** | | | |  | | 524.000^b^ | | 0.900 |
| ≤ 3 cigs/day | | 19 (82.6) | | 4 (17.4) | |  | |  |
| >3 cigs/day | | 35 (81.4) | | 8 (18.6) | |  | |  |
| Md(Q1~Q3) | | 3(2~5) | | 3(3~4) | |  | |  |
| **Smokeless Tobacco** | | | |  | | 0.192 | | 0.661 |
| Never or former consumer | | 148 (47.7) | | 162 (52.3) | |  | |  |
| Current consumer | | 36 (45.0) | | 44 (55.0) | |  | |  |
| **Frequency of Smokeless Tobacco** | | |  | | 0.745 | | 0.572^#^ | |
| Sometimes or Ad hoc | 2 (66.7) | | 1 (33.3) | |  | |  | |
| Daily | 32 (41.6) | | 45 (58.4) | |  | |  | |
| **Secondhand smoke(ire)** | | |  | | 0.877 | | 0.349 | |
| No | 114 (45.4) | | 137 (54.6) | |  | |  |  |
| Yes | 70 (50.4) | | 69 (49.6) | |  | |  |  |
| **Duration of Secondhand smoke(ire)** | | |  | | 2244.000^b^ | | 0.715 | |
| < 1hr/week | 22 (50.0) | | 22 (50.0) | |  | |  |  |
| ≥ 1hr/week | 46 (50.0) | | 46 (50.0) | |  | |  |  |
| Md(Q1~Q3) | 2(1~2) | | 2(1~2) | |  | |  |  |
| **Consumption of Fruits and Vegetables** | | |  | | 2.044 | | 0.153 | |
| Low(≤1.3servings/day) | 91 (51.1) | | 87 (48.9) | |  | |  |  |
| High(>1.3servings/day) | 93 (43.9) | | 119 (56.1) | |  | |  |  |
| **BP Status** |  | |  | | 10.010 | | **0.002**** | |
| Normotensive | 114 (41.9) | | 158 (58.1) | |  | |  | |
| Hypertensive | 70 (59.3) | | 48 (40.7) | |  | |  | |
| **BMI Status** |  | |  | | 12.993 | | **0.002**** | |
| Underweight | 13 (38.2) | | 21 (61.8) | |  | |  | |
| Normal | 81 (40.1) | | 121 (59.9) | |  | |  | |
| Overweight and obese | 90 (58.4) | | 64 (41.6) | |  | |  | |

| **Body Fat Status** |  |  | 5.604 | **0.018*** |
| --- | --- | --- | --- | --- |
| Normal | 98 (42.2) | 134 (57.8) |  |  |
| Excess | 86 (54.4) | 72 (45.6) |  |  |
| **Presence of CVDs risk factors** | |  | 20.328 | **<0.001***** |
| 0 risk factor | 9 (19.6) | 37 (80.4) |  |  |
| 1 risk factor | 43 (43.9) | 55 (56.1) |  |  |
| 2 risk factors | 62 (49.6) | 63 (50.4) |  |  |
| ≥ 3 risk factors | 70 (57.9) | 51(42.7) |  |  |
| **BMR** |  |  | 3.346 | 0.168 |
| Lower | 123 (44.2) | 155(55.8) |  |  |
| Ideal | 61 (54.5) | 51 (45.5) |  |  |
| **HGS** | |  | 1.857 | 0.174 |
| Lower | 61 (42.7) | 82 (57.3) |  |  |
| Ideal | 123 (49.8) | 124 (50.2) |  |  |

Significant at *p < 0.05; **p< 0.01; ***p< 0.001; ^#^ Fisher’s Exact Test; ^@^ formal education-up to master’s level and above; ^b^ Maan Whitney U test statistic

S1.Table 2: Factors associated with anxiety symptoms

| **Variable** | **Anxiety** **Symptoms** | | **χ²** | **p-value** |
| --- | --- | --- | --- | --- |
|  | **Presence [243 (62.3%)]** | **Absence [147 (37.7%)]** |  |  |
| **Socio-demographic** |  |  |  |  |
| **Age** |  |  | 17051.500^b^ | 0.437 |
| 30-39 years | 68 (58.6) | 48 (41.4) |  |  |
| 40-49 years | 53 (60.9) | 34 (39.1) |  |  |
| 50-59 years | 51 (70.8) | 21 (29.2) |  |  |
| 60+ years | 71 (61.7) | 44 (38.3) |  |  |
| Md(Q1~Q3) | 50(37~60) | 46(35~64) |  |  |
| **Sex** |  |  | 4.567 | **0.033*** |
| Male | 90 (69.8) | 39 (30.2) |  |  |
| Female | 160 (61.3) | 101 (38.7) |  |  |
| **Ethnicity** |  |  | 4.038 | **0.044*** |
| Brahmin/Chhetri | 196 (60.1) | 130 (39.9) |  |  |
| Dalit, Janajati and Madhesi | 47 (73.4) | 17 (26.6) |  |  |
| **Marital status** |  |  | 0.024 | 0.876 |
| Never married | 2 (66.7) | 1 (33.3) |  |  |
| Ever Married | 241 (62.3) | 146 (37.7) |  |  |
| **Education Status**  Illiterate  Literate^@^ | 40 (53.3)  203 (64.4) | 35 (46.7)  112 (35.6) | 3.184 | 0.074 |

| **Occupation** |  |  | 0.324 | 0.851 |
| --- | --- | --- | --- | --- |
| Unemployed | 55 (60.4) | 36 (39.6) |  |  |
| Housekeeper, Farmer and Labor | 159 (63.3) | 92 (36.7) |  |  |
| Private employee, Government job and Business | 29 (60.4) | 19 (39.6) |  |  |
| **Monthly family income** |  | | 16560.000^b^ | 0.299 |
| ≤ 30000 NPR | 183 (64.7) | 100 (35.3) |  |  |
| >30000 NPR | 60 (56.1) | 47 (43.9) |  |  |
| Md(Q1~Q3) | 30000(25000~35000) | 25000(25000~40000) |  |  |
| **CVDs Risk Factors**  **Smoking status** |  |  | 6.119 | **0.013*** |
| Never or former smoker | 193 (59.6) | 131 (40.4) |  |  |
| Current smoker | 50 (75.8) | 16 (24.2) |  |  |
| **Number of cigarettes/days** |  | | 386.000^b^ | 0.800 |
| ≤ 3 cigs/day | 33 (76.7) | 10 (23.3) |  |  |
| >3 cigs/day | 17 (73.9) | 6 (26.1) |  |  |
| Md(Q1~Q3) | 3(2~6) | 3(2~4) |  |  |
| **Smokeless Tobacco** |  |  | 11.586 | **0.001**** |
| Never or former consumer | 180 (58.1) | 130 (41.9) |  |  |
| Current consumer | 63 (78.8) | 17 (21.3) |  |  |
| **Frequency of Smokeless Tobacco** | | | 0.272 | 0.517^#^ |
| Sometimes or Ad hoc | 1 (33.3) | 2 (66.7) |  |  |
| Daily | 16 (20.8) | 61 (79.2) |  |  |

| **Secondhand smoke(ire)** | |  | | | | 16.101 | | **<0.001***** | |
| --- | --- | --- | --- | --- | --- | --- | --- | --- | --- |
| No | | 138 (55.0) | | 113 (44.0) | |  | |  | |
| Yes | | 105 (75.5) | | 34 (24.5) | |  | |  | |
| **Duration of Secondhand smoke(ire)** | | | | | | 1530.000^b^ | | 0.204 | |
| < 1hr/week | | 30 (68.2) | | 14 (31.8) | |  | |  | |
| ≥ 1hr/week | | 72 (78.3) | | 20 (21.7) | |  | |  | |
| Md(Q1~Q3) | | 2(1~2) | | 2(1~2) | |  | |  | |
| **Consumption of Fruits and Vegetables** | | | | | | 0.053 | | 0.819 | |
| Low(≤1.3servings/day) | | 112 (62.9) | | 66 (37.1) | |  | |  | |
| High(>1.3servings/day) | | 131 (61.8) | | 81 (38.2) | |  | |  | |
| **BP Status** | |  | |  | | 9.397 | | **0.002*** | |
| Normotensive | | 156 (57.4) | | 116 (42.6) | |  | |  | |
| Hypertensive | | 87 (73.7) | | 31 (26.3) | |  | |  | |
| **BMI Status** | |  | |  | | 23.297 | | **<0.001***** | |
| Underweight | | 11 (32.4) | | 23 (67.6) | |  | |  | |
| Normal | | 118 (58.4) | | 84 (41.6) | |  | |  | |
| Overweight and obese | | 114 (74.0) | | 40 (26.0) | |  | |  | |
| **Body Fat Status** | |  | |  | | 21.046 | | **<0.001***** | |
| Normal | | 123 (53.0) | | 109 (47.0) | |  | |  | |
| Excess | | 120 (75.9) | | 38 (24.1) | |  | |  | |
| **Presence of CVDs risk factors** | | | | | 47.291 | | **<0.001***** | |  |
| 0 risk factor | 10 (21.7) | | 36 (78.3) | |  | |  | |  |
| 1 risk factor | 55 (56.1) | | 43 (43.9) | |  | |  | |  |
| 2 risk factors | 84 (67.2) | | 41 (32.8) | |  | |  | |  |
| ≥ 3 risk factors | 94 (77.7) | | 27 (22.3) | |  | |  | |  |
| **BMR** |  | |  | | 2.777 | | 0.097 | |  |
| Lower | 166 (59.7) | | 112(40.3) | |  | |  | |  |
| Ideal | 77 (68.8) | | 35 (31.3) | |  | |  | |  |
| **HGS** |  | |  | | 4.796 | | **0.029*** | |  |
| Lower | 79 (55.2) | | 64 (44.8) | |  | |  | |  |
| Ideal | 164 (66.4) | | 83 (33.6) | |  | |  | |  |

Significant at *p < 0.05; **p< 0.01; ***p< 0.001; ^#^ Fisher’s Exact Test; ^@^ formal education-up to master’s level and above; ^b^ Maan Whitney U test statistic

S1. Table 3: Factors associated with Stress Symptoms

| **Variable** | **Stress Symptoms** | | **χ²** | **p-value** |
| --- | --- | --- | --- | --- |
|  | **Presence [215 (55.1%)]** | **Absence [175 (44.9%)]** |  |  |
| **Socio-demographic** |  |  |  |  |
| **Age** |  |  | 18055.000^b^ | 0.478 |
| 30-39 years | 66 (56.9) | 50 (43.1) |  |  |
| 40-49 years | 50 (57.5) | 37 (42.5) |  |  |
| 50-59 years | 38 (52.8) | 34 (47.2) |  |  |
| 60+ years | 61 (53.0) | 54 (47.0) |  |  |
| Md(Q1~Q3) | 47(36~60) | 50(36~61) |  |  |
| **Sex** |  |  | 4.575 | **0.032*** |
| Male | 81 (62.8) | 48 (37.2) |  |  |
| Female | 134 (51.3) | 127 (48.7) |  |  |
| **Ethnicity** |  |  | 4.501 | **0.034*** |
| Brahmin/Chhetri | 172 (52.8) | 154 (47.2) |  |  |
| Dalit, Janajati and Madhesi | 43 (67.2) | 21 (32.8) |  |  |
| **Marital Status** |  |  | 0.581 | 0.446 |
| Never married | 1 (33.3) | 2 (66.7) |  |  |
| Ever Married | 214 (55.3) | 173 (44.7) |  |  |
| **Education Status**  Illiterate  Literate^@^ | 36 (48.0)  179 (56.8) | 39 (52.0)  136 (43.2) | 1.907 | 0.168 |

| **Occupation** | |  | |  | | 3.385 | | 0.184 |  |
| --- | --- | --- | --- | --- | --- | --- | --- | --- | --- |
| Unemployed | | 46 (50.2) | | 45 (49.5) | |  | |  |  |
| Housekeeper, Farmer and Labor | | 137 (54.6) | | 114 (45.4) | |  | |  |  |
| Private employee, Government job and Business | | 32 (66.7) | | 16 (33.3) | |  | |  |  |
| **Monthly family income** | | | | | | 18620.000^b^ | | 0.822 |  |
| ≤ 30000 NPR | | 157 (55.5) | | 126 (44.5) | |  | |  |  |
| >30000 NPR | | 58 (54.2) | | 49 (45.8) | |  | |  |  |
| Md(Q1~Q3) | | 30000(25000~30000) | | 30000(25000~40000) | |  | |  |  |
| **CVDs Risk Factors**  **Smoking status** | |  | |  | | 1.571 | | 0.129 |  |
| Never or former smoker | | 174 (53.7) | | 150 (46.3) | |  | |  |  |
| Current smoker | | 41 (62.1) | | 25 (37.9) | |  | |  |  |
| **Number of cigarettes/days** | | | | | | 384.000 | | 0.053 |  |
| ≤ 3 cigs/day | | 30 (69.8) | | 13 (30.2) | |  | |  |  |
| >3 cigs/day | | 11 (47.8) | | 12 (52.2) | |  | |  |  |
| Md(Q1~Q3) | | 3(2~5) | | 3(2~4) | |  | |  |  |
| **Smokeless Tobacco** | |  | |  | | 4.028 | | **0.048*** |  |
| Never or former consumer | | 164 (52.9) | | 146 (47.1) | |  | |  |  |
| Current consumer | | 51 (63.7) | | 29 (36.3) | |  | |  |  |
| **Frequency of Smokeless Tobacco** | | | | | | 0.011 | | 1.000^#^ |  |
| Sometimes or Ad hoc | | 2 (66.7) | | 1 (33.3) | |  | |  |  |
| Daily | | 49 (63.6) | | 28 (36.4) | |  | |  |  |
| **Secondhand smoke(ire)** |  | | | | 6.917 | | **0.009**** | | |
| No | 126 (50.2) | | 125 (49.8) | |  | |  | | |
| Yes | 89 (64.0) | | 50 (36.0) | |  | |  | | |
| **Duration of Secondhand smoke(ire)** | | | | | 1849.500^b^ | | 0.115 | | |
| < 1hr/week | 24 (54.5) | | 20 (45.5) | |  | |  | | |
| ≥ 1hr/week | 61 (66.3) | | 31 (33.7) | |  | |  | | |
| Md(Q1~Q3) | 2(1~2) | | 1(1~2) | |  | |  | | |
| **Consumption of Fruits and Vegetables** | | | | | 0.032 | | 0.859 | | |
| Low(≤1.3servings/day) | 99 (55.6) | | 79 (44.4) | |  | |  | | |
| High(>1.3servings/day) | 116 (54.7) | | 96 (45.3) | |  | |  | | |
| **BP Status** |  | |  | | 15.825 | | **<0.001***** | | |
| Normotensive | 132 (48.5) | | 140 (51.5) | |  | |  | | |
| Hypertensive | 83 (70.3) | | 35 (29.7) | |  | |  | | |
| **BMI Status** |  | |  | | 44.488 | | **<0.001***** | | |
| Underweight | 9 (26.5) | | 25 (73.5) | |  | |  | | |
| Normal | 88 (43.6) | | 114 (56.4) | |  | |  | | |
| Overweight and obese | 118 (76.6) | | 36 (23.4) | |  | |  | | |
| **Body Fat Status** |  | |  | | 43.761 | | **<0.001***** | | |
| Normal | 96 (41.4) | | 136 (58.6) | |  | |  | | |
| Excess | 119 (75.3) | | 39 (24.7) | |  | |  | | |

| **Presence of CVDs risk factors** | | | 62.936 | **<0.001***** |
| --- | --- | --- | --- | --- |
| 0 risk factor | 6 (13.0) | 40 (87.0) |  |  |
| 1 risk factor | 41 (41.8) | 57 (58.2) |  |  |
| 2 risk factors | 76 (60.8) | 49 (39.2) |  |  |
| ≥ 3 risk factors | 92 (76.0) | 29 (24.0) |  |  |
| **Anthropometric measurements** | | |  |  |
| **BMR** |  |  | 20.777 | **<0.001***** |
| Lower | 133 (47.8) | 145(52.2) |  |  |
| Ideal | 82 (73.2) | 30 (26.8) |  |  |
| **HGS** |  |  | 9.527 | **0.002**** |
| Lower | 63 (44.1) | 80 (55.9) |  |  |
| Ideal | 152 (61.5) | 95 (38.5) |  |  |

Significant at *p < 0.05; **p< 0.01; ***p< 0.001; ^#^ Fisher’s Exact Test; ^@^ formal education-up to master’s level and above; ^b^ Maan Whitney U test statistic
